# The Non-Fibrillar Side of Fibrosis: Contribution of the Basement Membrane, Proteoglycans, and Glycoproteins to Myocardial Fibrosis

**DOI:** 10.3390/jcdd6040035

**Published:** 2019-09-23

**Authors:** Michael Chute, Preetinder Aujla, Sayantan Jana, Zamaneh Kassiri

**Affiliations:** Department of Physiology, Cardiovascular Research Center, University of Alberta, Edmonton, AB T6G 2S2, Canada; Mchute@ualberta.ca (M.C.); pkaujla@ualberta.ca (P.A.); sjana@ualberta.ca (S.J.)

**Keywords:** heart, fibrosis, remodeling, extracellular matrix, basement membrane, proteoglycans

## Abstract

The extracellular matrix (ECM) provides structural support and a microenvironmentfor soluble extracellular molecules. ECM is comprised of numerous proteins which can be broadly classified as fibrillar (collagen types I and III) and non-fibrillar (basement membrane, proteoglycans, and glycoproteins). The basement membrane provides an interface between the cardiomyocytes and the fibrillar ECM, while proteoglycans sequester soluble growth factors and cytokines. Myocardial fibrosis was originally only linked to accumulation of fibrillar collagens, but is now recognized as the expansion of the ECM including the non-fibrillar ECM proteins. Myocardial fibrosis can be reparative to replace the lost myocardium (e.g., ischemic injury or myocardial infarction), or can be reactive resulting from pathological activity of fibroblasts (e.g., dilated or hypertrophic cardiomyopathy). Contribution of fibrillar collagens to fibrosis is well studied, but the role of the non-fibrillar ECM proteins has remained less explored. In this article, we provide an overview of the contribution of the non-fibrillar components of the extracellular space of the heart to highlight the potential significance of these molecules in fibrosis, with direct evidence for some, although not all of these molecules in their direct contribution to fibrosis.

## 1. Introduction

All cardiomyopathies, regardless of the initiating cause, involve myocardial remodeling with myocardial fibrosis as one of the key characteristics [1,2]. Fibrosis refers to expansion of the extracellular matrix (ECM) and accumulation of ECM proteins, and is broadly classified as reactive or reparative fibrosis. In reactive fibrosis, aberrant activation of fibroblasts results in excess production and deposition of ECM proteins in the myocardium expanding the interstitial space. This can be brought about by a range of pathological stimuli such as hypertension, pressure overload, aging, and diabetes. On the other hand, reparative or replacement fibrosis refers to the process of scar formation in areas of myocyte loss due to necrosis. Reparative fibrosis is typically the result of an ischemic injury or myocardial infarction and serves as a ‘patch’ to close the gap that would otherwise exist at the site of myocyte loss [3]. Fibrosis adversely impacts cardiac function as it reduces myocardial compliance imposing diastolic dysfunction which can progress to diastolic heart failure even in the absence of systolic dysfunction. This is known as heart failure with preserved ejection fraction (HFpEF). Although the role of fibrosis in HFpEF has become increasingly well-recognized, it is important to note that fibrosis can also contribute to systolic dysfunction as it can replace the contracting cardiomyocytes, thereby compromising cardiac contractility. Therefore, fibrosis is a key characteristic of heart failure with reduced ejection fraction (HFrEF) as well as HFpEF.

In addition to the fibrillar proteins such as collagen (Table 1), a significant fraction of the myocardial ECM is comprised of non-fibrillar proteins (Table 2). These include the basement membrane proteins, proteoglycans, glycoproteins, and glycosaminoglycans. The ECM is an extraordinary component of the myocardial tissue that provides structural support to preserve cardiac geometry and facilitates force transmission, while the non-fibrillar ECM provides an interstitial storage space to retain numerous growth factors and cytokines in their latent form until released in response to a physiological or a pathological cue [4]. The interaction between the ECM and cardiomyocytes is through a collagen–integrin–cytoskeleton–myofibril link that is important in transducing extracellular signals to regulate cardiomyocyte function. Traditionally, fibrosis was only attributed to accumulation of fibrillar collagens (collagen types I and III), however it is becoming increasingly evident that remodeling of the non-fibrillar ECM is also an important contributor to formation of fibrotic lesions. Here, we will provide an overview of the non-fibrillar ECM proteins, their reported or potential involvement in myocardial fibrosis. However, it is important to note that a lack of information on the role of any non-fibrillar ECM protein could be mainly due to deficiency of research in this field rather than their lack of contribution to cardiac fibrosis.

## 2. Fibrillar ECM in the Myocardium

The fibrillar ECM network is the most commonly investigated part of the ECM and is primarily comprised of fibrillar collagens. Based on their structure, collagen molecules are divided into two main classes: Fibril forming collagens which include collagen type I, type II, type III, type V, and type XI; and non-fibril forming collagens, collagen type IV and VI [74,75]. Collagen type I forms thick rod-like fibers and underlies the myocardial tensile strength, while collagen type III forms fine network of fibers and accounts for its distensibility [76], collagen II is expressed mainly in the cartilage and type V in dermal tissue. Fibrillar collagens are produced as triple helix pro-collagens that are secreted to the extracellular interstitium for post-translational modifications such as enzymatic removal of the loose N- and C-propeptides, crosslinking and stabilization of their fibrillar structure [6,74,77,78]. The C-terminal and N-terminal propeptides of procollagens (PICP, PINP for type I; PIIICP and PIIINP for type III) are released during biosynthesis of these collagen fibrils and have been considered as biomarkers of collagen synthesis [6]. Subsequently, hydroxylation and oxidative deamination of collagens by lysyl hydroxylase (PLOD1) and lysyl oxidase (LOX) lead to cross-linking and stabilization of collagen fibers [79,80]. Further post-translational regulation of the collagen fibers is mediated by matricellular proteins, the non-ECM proteins that reside in the interstitial space and play important roles in stabilization of collagen fibers; these include SPARC, osteopontin, and periostin [81,82]. Contribution of the fibrillar ECM to myocardial fibrosis has been extensively reviewed [4,6,83,84] (Table 2), and as such will not be the main focus of this article.

## 3. Basement Membrane Proteins in Myocardial Fibrosis

The basement membrane in the myocardium is a specialized form of ECM, a pericellular ECM that serves as the interface between the cardiomyocyte and the interstitial ECM [85,86]. The basement membrane consists primarily of fibronectin, laminin, collagen type IV, and basement membrane proteoglycans (discussed in Section 5, under Proteoglycans) [87].

### 3.1. Fibronectin (FN) Is a Dimeric Glycoprotein in the ECM

FN mediates the connection between the cells and the interstitial ECM by binding to cell membrane receptor integrins, and other ECM proteins such as collagens, fibrin, and heparan sulfate proteoglycans such as syndecans. In vertebrates, two types of FN are present, soluble plasma FN and insoluble cellular FN. FN is produced from a single gene, but alternative splicing of its mRNA leads to formation of different isoforms. Alternatively-spliced domain A (EDA) fibronectin (EDA-FN) has been associated with fibroblast differentiation [17]. EDA-FN increases α-smooth muscle actin (αSMA) expression, collagen deposition, cell contractility, and focal adhesion kinase (FAK) activation through its phosphorylation. Blocking of integrin α_4_β_7_ reduced fibroblast adhesion to EDA-FN, and the EDA segment itself was found to be sufficient to induce fibroblast differentiation. EDA-FN is proposed to act through the MAPK-ERK1/2 pathway as inhibition of MEK1/2 inhibited EDA-FN-induced αSMA expression, and transforming growth factor β1 (TGFβ1) is unable to induce αSMA upregulation on non-adherent cells [88]. Inhibition of FAK/Src kinase activity blocks TGFβ1-induced myofibroblast differentiation through phosphorylation of FAK, which is a requirement for myofibroblast differentiation. It has been reported that fibroblasts cultured on FN-coated plates have the greatest increase in αSMA expression. However, it has also been reported that FN alone induces fibroblast migration and not myofibroblast differentiation. In the presence of both TGFβ1 and FN, proliferation of fibroblasts increased, while TGFβ1 alone enhanced COL1α1, EDA-FN expression, and reduced cell migration [89].

Following acute myocardial infarction in patients, FN deposition is increased in the infarcted area [90]. In a mouse model of ischemia/reperfusion injury, inhibition of FN polymerization inhibited myofibroblast activation and subsequent fibrotic deposition [91]. Genetic deletion of FN in adult age (Fn^flox/flox^/CMV-Cre), reduced the pressure overload-induced cardiac hypertrophy and fibrosis [92]. Similarly, FN deletion in fibroblasts (Fn^flox/flox^/Periostin-Cre) resulted in cardioprotective effects and reduced fibrosis following ischemia-reperfusion [91]. FN has also been identified to play an important role in the maturation, stabilization, and deposition of collagen I [18], as well as mediating the processing of 45 kDa pro-LOX to the enzymatically activate 30 kDa LOX perhaps by providing a scaffold to facilitate this process [93,94]. In summary, FN promotes the transition of fibroblasts to myofibroblasts and facilitates the deposition and cross-linking of collagen fibers in fibrotic areas.

### 3.2. Laminin Is One of the Main Proteins in the Basement Membrane

The laminin family of proteins consists of 3 chains, α, β, and γ which are assembled in a branched structure containing short arms and a coiled-coil long arm with a globular domain [95]. Laminins are responsible for the organization of the basement membrane on cell surfaces. The short arms of laminins are the key structures that allow them to assemble the basement membrane independently of other basement membrane components. The short arms are the N-terminal portion of the α, β, and γ chains; laminin polymers are formed by the interaction of the short arm laminin N-terminal domains of adjacent laminins [96,97,98]. Perlecan and nidogen are proteoglycans that connect the laminin polymers to Col IV polymers for increased basement membrane stability [99,100,101,102]. The long arm of laminin is a triple-strand helix of the α, β, and γ chains woven together and stabilized by disulfide bonds [97,100]. Extending out from this triple helix coil is the C-terminal laminin globular domain that is responsible for cell surface binding to integrin and α-dystroglycan [103,104,105]. Laminin-integrin interactions through this globular domain activate signals important in cellular functions [105]. Targeted tissue-specific genetic deletion of laminin eliminates the formation of the basement membrane in that tissue [106,107]. Although there is no report on the direct contribution of laminin to fibrosis, laminin appears to contribute to other aspects of cardiovascular remodeling. Mice deficient in laminin alpha 4 (LAMA4) display endothelial dysfunction, dilated vessels, hemorrhages, as well as cardiac hypertrophy progressing to heart failure due to a loss of connection between the basement membrane and actin cytoskeleton, and between the cardiomyocyte and the ECM resulting in apoptosis [108]. In general, the N-terminus of laminins interacts with interstitial ECM proteins and provides assembly and stability to the basement membrane; while the C-terminus of laminins interacts with the cell surface receptors and mediates adhesion/migration, survival/apoptosis, signaling, differentiation, and gene expression [109,110].

### 3.3. Collagen IV Forms a Network with Laminin in the Basement Membrane

Col IV forms a covalently-stabilized network through three types of self-assembly and binding to laminin [102]. Firstly, the N-terminal domains of Col IV and laminin interact spontaneously to form dimeric and trimeric intermediates [111,112] while LOX and disulfide-derived covalent cross-links further stabilize the structure [112]. Secondly, the self-interactions of the globular C-terminal domain of laminin extend the Col IV network. Specificity of this collagenous chain assembly is dependent on C-terminal subunits of laminin [113,114]. Finally, parallel collagen IV filaments interact with each other to form a network [111,115]. It is proposed that these interactions drive network complexity as parallel collagen IV interactions were not observed in the absence of N- and C- terminal interactions [111].

During embryogenesis, Col IV does not organize the basement membrane assembly [116]. Mice deficient in the major Col IV isoform α1(IV)_2_α2(IV) developed normally up to embryonic day 9.5 (ED9.5), and basement membranes were assembled, indicating that this isoform was not necessary for basement membrane deposition and assembly. Lethality occurred from ED10.5 to ED11.5 due to structural deficiencies in the basement membrane resulting in a heart that was unable to cope with an increase in pressure during cardiogenesis [116]. These data support the notion that Col IV is necessary for the structural integrity of the basement membrane during mechanical stress, but is nonessential during the initial assembly of the basement membrane components. Expression of *COL4A1* and *COL4A2* was found to be increased in the infarcted regions of rats following MI [117]. In patients with hypertrophic cardiomyopathy, increased collagen IV correlated with systolic and diastolic dysfunctions [118].

## 4. Proteoglycans and Glycoproteins in Fibrosis

### 4.1. Proteoglycans and Glycosaminoglycans

**Figure 1:**
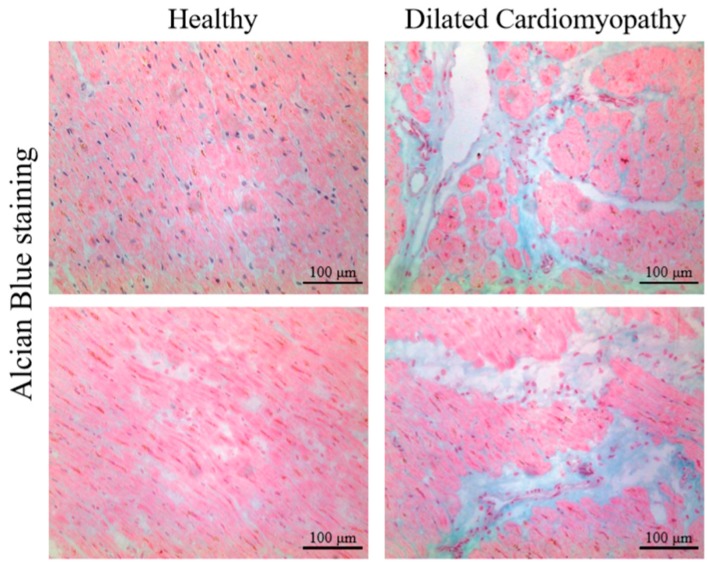
Alcian blue staining showing areas of glycosaminoglycan (GAG) accumulation in hearts from patients with dilated cardiomyopathy (DCM) compared to healthy (non-failing control, NFC) hearts.

Proteoglycans are glycosylated proteins that consist of a core protein with covalently attached linear polysaccharides, glycosaminoglycan (GAG) chains. Accumulation of GAGs in the fibrotic areas can be visualized by Alcian Blue staining (Figure 1). The GAG chains are linear carbohydrate polymers (polysaccharides) with repeating disaccharide units. Polysaccharide chains are too stiff to fold up into the compact structures of most other polypeptides, therefore GAGs form highly extended conformations that occupy a large volume relative to their mass. While there is no accurate estimate of what fraction of the ECM is occupied by the non-fibrillar versus fibrillar proteins, this ratio varies by tissue type. For instance, in connective tissue, GAGs are estimated to occupy less than 10% weight of the fibrous ECM proteins, but occupy 80–90% of the extracellular space [119].

GAGs are highly negatively charged and as such able to interact with positively charged molecules such as other ECM proteins, cytokines, chemokines, pathogens, growth factors and proteases. Through this function of GAGs, the ECM is able to serve as a reservoir for soluble growth factors and cytokines by sequestering them in the interstitial space until they need to be released, or made accessible to their receptors, in response to physiological or pathological cues [4]. Heparan sulphate is a GAG found on many proteoglycans in the heart, and the anticoagulant heparin is the naturally occurring heparan sulfate GAG produced by mast cells, which crucial to its function, it carries the highest negative charge density of any known biological molecule [120,121]. GAGs are a major determinant of the function of the proteoglycans. In explanted failing hearts from adult and pediatric patients, the affinity of GAGs for TGFβ1 is reduced in adult compared to pediatric failing hearts, resulting in significantly greater fibrosis in the adult compared to pediatric failing hearts [50].

**Figure 2:**
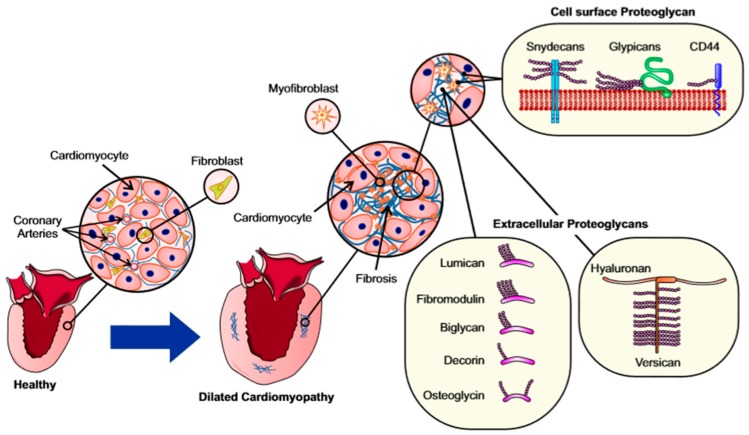
Accumulation of proteoglycans in addition to the fibrillar collagens in myocardial fibrosis. Myocardial remodeling in dilated cardiomyopathy entails enlargement of cardiomyocytes, increased amount of extracellular matrix and fibrosis. The proteoglycans present in fibrotic lesions can be extracellular or membrane-bound.

Proteoglycans make up a significant fraction of a scar or fibrotic lesion (Figure 2). Proteoglycans are divided into four groups based on their extracellular localization, size and structural properties: cell surface or membrane-bound proteoglycan (Syndecan, Glypicans, CD44), extracellular proteoglycans (Versican, Aggrecan, Neurocan, Brevican), basement membrane proteoglycans (Perlecan, Collagen type XVIII, Agrin), and small leucine rich proteoglycans (SLRPs, such as Biglycans, Decorin, Lumican, Fibromodulin, and Osteoglycin). A thorough review on proteoglycan families, structures and nomenclatures has been published by Iozzo and Shaefer [122]. Here, we will focus on the proteoglycans relevant to cardiac remodeling and fibrosis.

### 4.2. Cell Surface Proteoglycans

Syndecans and glypicans are the two main families of cell surface proteoglycans found in mammals [122]. Syndecans are transmembrane proteins with a cytoplasmic tail, a transmembrane spanning domain, and an extracellular domain with chondroitin or heparan sulfate GAG chains. Glypicans are anchored to the cell surface by glycosylphosphatidylinositol (GPI). Expression of all four syndecans has been reported to increase following myocardial infarction [20]. Roles of syndecan-1 and -4 have been explored in myocardial fibrosis and have been found to be crucial for “wound healing” and scar formation following myocardial infarctions [21,123]. In a model of MI, syndecan-1 knockout mice exhibited increased left ventricular rupture and dilation due to reduced collagen upregulation and impaired cross-linking [21,24,25,124]. Syndecan-1 overexpression, on the other hand, improved collagen matrix formation and protected against rupture post-MI. Interestingly, overexpression of syndecan-1 in rats reduced the post-MI fibrosis by suppressing inflammation and inhibiting the p38 MAPK pathway [125]. Syndecan-1 was also found to be essential in angiotensin II (Ang II)-induced cardiac fibrosis, and upregulated in Ang II-induced fibrosis [19]. Syndecan-1 deficiency, in vivo and in fibroblasts, blunted the Ang II-induced rise in expression of CTGF, collagen type I, and Smad2 phosphorylation as well as myocardial fibrosis [19]. In humans, plasma syndecan-1 levels correlate with fibrosis biomarkers, and increased syndecan-1 was associated with an increased risk of the primary outcome in heart failure patients with preserved ejection fraction but not those with reduced ejection fraction [126]. These reports collectively support and important role of syndecan-1 in ECM remodeling through regulation of pro-fibrotic signaling pathways.

Syndecan-4 is found at focal adhesions, the site of mechanotransduction signaling [22], and has been reported to contribute to myofibroblast differentiation following pressure overload or MI. Cardiac fibroblasts isolated from syndecan-4 knockout mice have reduced signaling of molecules associated with mechano-transduction such as focal adhesion kinase (FAK), protein kinase B, and RhoA [124]. In response to mechanical stress, the cytoplasmic tail of syndecan-4 is dephosphorylated and the calcineurin-NFAT signaling is activated, resulting in myofibroblast differentiation and collagen expression [23,24]. Syndecan-4 has also been shown to attenuate the Ca^2+^ permeable TRPC7 channel, an activator of the calcineurin-NFAT pathway [127] that is also necessary for TGFβ mediated myofibroblast differentiation [128]. In pressure overload, syndecan-4 knockout mice were found to have attenuated myocardial stiffness [25]. This was attributed to the reduced expression of LOX. Electron microscopy identified the extracellular domain of syndecan-4 as having a direct role in facilitating LOX collagen cross-linking activity [25]. Syndecan-1 knockout mice had similar results in a pressure overload model, but this was attributed to reduced tissue transglutaminase activity (another collagen cross-linker) [21].

Very little is known about the effects of glypicans on myocardial fibrosis [129,130]. Glypican-6 (GPC6) is produced by both cardiac fibroblasts and cardiomyocytes, and was found to be upregulated in the heart following cardiac pressure overload mice [26]. Overexpression of GPC6 in cardiomyocytes increased hypertrophic growth and ERK1/2 signaling [26]. GPC6 expression in cardiac fibroblasts is increased in response to Ang II and BMP4. GPC6 also regulates fibroblast growth factor (FGF) signaling. This supports GPC6 having a role in ECM remodeling, however in vitro studies could not show GPC6 having an effect on cardiac fibroblast collagen expression, proliferation, or migration [26].

### 4.3. Extracellular Proteoglycans (Hyalectins)

Hyalectins are a group of large proteoglycans that can bind hyaluronan (by their N-terminal domain) and lectins (by their C-terminal domain), and contain numerous and long GAG chains that interact with structural and non-structural proteins [122]. This group of proteoglycans include versican, neurocan, aggrecan. and brevican; and are sometimes presented in the same category as SLRPs as they are all extracellular proteoglycans. Hyalectins control tissue permeability, hygroscopicity (ability to retain water) and compressibility. A number of studies have proposed the contribution of versican in ECM remodeling and heart disease, but the role of aggrecan (a cartilage proteoglycan with potential role in vascular stiffness [131]), neurocan (specific to neuronal tissue [132,133,134]), and brevican in heart disease remains unexplored.

Versican serves as a determinant of extracellular volume [135,136], has been found to be essential for normal cardiac development, and is produced by cardiac fibroblasts and cardiomyocytes [27,28]. Versican knockout mice display a high rate of embryonic lethality due to failed cardiac development at ED 9.5, and surviving mice have ventricular septum defects because of the key role of versican in the atrioventricular cushion and ventricular septa for septum fusion. In cardiac pressure overload, levels of verscian, and versicanase ADAMTS4, increased along with p150 versican fragment levels [29]. Interestingly, inhibition of ADAMTS4 by pentosan polyphosphate (PPS), reduced versican fragment levels and improved systolic function [29]. The p150 versican fragment is associated with myocardial edema, a feature of heart failure. Versican expression and cleavage was also increased following stimulation of cardiac cells with cytokines associated with heart failure, indicating inflammation as one of the regulators for versican homeostasis [30].

### 4.4. Basement Membrane Proteoglycans

Agrin and perlecan are two heparin sulfate proteoglycans that are found in the basement membrane [137]. Agrin binds to α-dystroglycan, integrins, and laminins [138,139,140,141]. The ability of agrin to bind both α-dystroglycan and laminin may facilitate increased laminin binding to cell surfaces. Perlecan binds to nidogen-1, integrin α2β1, and α-dystroglycan [142,143,144]. The heparin sulfate chains have the ability to bind to laminin as well as Col IV [143]. Similar to agrin, this binding pattern could improve laminin binding to cell surfaces and increase stability in the myocardium. Experiments using perlecan-deficient mice found that basement membranes lacking perlecan deteriorated in the heart, which lead to cell-cell detachment and blood leakage into the pericardial cavity during embryogenic development [145]. Sarcomere formation and myocyte function were not altered in perlecan-null embryonic hearts, but the heart showed mechanical stability with lower amounts of critical basement membrane components, Col IV and laminins, resulting in loss of the basement membrane despite intact adherens junctions. Mice with partial perlecan loss (perlecan^+/−^) showed increased susceptibility to cardiac dysfunction in a cryoinjury model [145]. These reports highlight the key role of perlecan in basement membrane assembly and stabilization.

Perlecan and agrin can bind to and sequester growth factors in the ECM through their heparin sulfate groups [32], serving as an extracellular storage facility. These growth factors include fibroblast growth factors (FGF), transforming growth factor β (TGFβ), bone morphogenetic proteins (BMP), vascular endothelial growth factors (VEGF) and heparan-binding epidermal growth factors (HB-EGF) [34,35,36,37,146]. Perlecan acts in concert with the receptor of these growth factors to promote binding [35,36]. For example, it induces neovascularization in a model of angiogenesis by promoting FGF2 binding to its receptor to induce the downstream effectors [37]. Following a myocardial infarction, perlecan has been found in the infarcted and border regions, and linked to fibrosis and angiogenesis in these regions [31], however, whether it directly contributes to myocardial fibrosis remains to be determined.

### 4.5. Small Leucine Rich Proteoglycans

The small leucine rich proteoglycan (SLRP) family are a group of secreted, low molecular weight proteoglycans. SLRPs affect collagen fibrillogenesis by binding collagen fibrils and regulating collagen fibril diameter and interfibrillar spacing in the ECM [147]. The main SLRPs reported to be involved in heart disease are biglycans, decorin, lumican, fibromodulin, and osteoglycin.

Biglycan and decorin share a high degree of homology among SLRPs and carry GAG chains of chondroitin or dermatan sulfate [122]. Biglycan is expressed in the healthy heart and its expression is increased following MI or pressure overload [38]. Biglycan has been found to co-localize with collagen fibers in the infarct scar [40], and its expression is increased in activated fibroblasts, but not in other cardiac cells following pressure overload [39]. In addition, biglycan knockout mice demonstrate reduced hypertrophy and cardiac fibrosis following pressure overload [39]. Following MI, biglycan-deficiency resulted in a high rate of mortality due to cardiac rupture, increased ventricular dilation, and impaired collagen matrix organization [40]. Biglycan can also stimulate innate immunity through Toll-like receptors (TLRs), which could mediate inflammation-mediated cardiac remodeling [147,148]. Overall, biglycan acts as a pro-fibrotic proteoglycan that stabilizes the collagen fibers, and as such its presence is essential during post-MI reparative fibrosis, but not in the cases of reactive fibrosis such as hypertrophic cardiomyopathy.

Decorin, like biglycan, is expressed in the healthy heart and is increased in myocardial fibrosis following MI or pressure overload [38]. In explanted hearts from patients with terminal HFrEF, decorin expression is increased [38], but not in patients with heart failure due to dilated cardiomyopathy [50]. Decorin acts to inhibit TGFβ1 bioactivity by decreasing expression and sequestration [41]. In vitro, decorin inhibits collagen expression following TGFβ1 administration in human cardiac fibroblasts [149]. Overexpression of decorin reduces fibrosis and improves cardiac function in hypertensive rats by inhibiting the TGFβ1-Smad pathway [42]. In contrast to biglycan and lumican, decorin has anti-fibrotic functions, possibly through sequestering TGFβ1 and reducing its bioavailablity, and therefore has received attention as a potential anti-fibrotic molecule.

Lumican can bind to collagen and is abundant in fibrotic tissues such as ischemic hearts [43]. Like the aforementioned SLRPs, lumican expression is increased in the hearts of HFrEF patients and pressure overloaded mice [38]. Expression of lumican was increased in vitro following mechanical stretch, or in response to inflammatory stimuli, IL-1β or lipopolysaccharide [38]. All three stimuli are important for ECM remodeling in the heart. Lumican knockout mice exhibit a lack of corneal transparency and increased skin fragility, with irregular collagen fibril contours and increased fibril diameter in cornea, skin, and tendons, indicating a crucial role of lumican in collagen processing and fiber formation [150]. Addition of recombinant lumican in vitro increased collagen I expression in cardiac fibroblasts [38]. Lumican has also been found to increase the expression of LOX. Although a pro-fibrotic function has been proposed for lumican, its expression was inversely correlated with degree of myocardial fibrosis in explanted failing hearts from patients [50]. Therefore, contribution of lumican to myocardial fibrosis may be complex.

### 4.6. Fibromodulin and Osteoglycin

Fibromodulin is another SLRP that binds to collagen and has been found in heart valves [151] and in the myocardium, and one of the proteoglycans that were upregulated in the mouse heart following pressure-overload [38]. However, its direct role in myocardial fibrosis has not yet been determined.

Osteoglycin, also known as mimecan, is critical for optimal collagen assembly in the diseased heart. Osteoglycin-deficient mice exhibit a high rate of left ventricular rupture following MI which was ameliorated by adenoviral overexpression of osteoglycin, as bridges were formed between collagen fibrils with osteoglycin, forming non-enzymatic collagen cross-links that stabilize the collagen fibers in the infarct scar tissue [152]. Studies on osteoglycin knockout mice further suggest a beneficial function for osteoglycin in preventing aging-related myocardial fibrosis and diastolic dysfunction [153]. A novel variant of osteoglycin (72 kDa) resulting from chondroitin sulfate GAG chain substitution of the core protein, has been reported to be expressed by immune cells in the heart following an inflammatory insult, and that this isoform can bind to and activate TLR4 [154]. This could place osteoglycin at the cross-talk between inflammation and fibrosis, making it an attractive target in treating various cardiomyopathies.

## 5. Glycoproteins

Proteoglycans are proteins that are heavily glycosylated. The basic proteoglycan unit consists of a ‘core protein’ with one or more covalently attached GAG chains. The extracellular glycoproteins include non-structural extracellular proteins such as thrombospondins, and matricellular proteins (SPARC, perisostin, tenasin).

Thrombospondins (TSPs) are a family of secreted calcium-binding glycoproteins [59], highly expressed during development but are almost undetectable in adults. TSP1 is able to activate TGFβ1 during the inflammatory stage of MI, allowing fibroblasts to differentiate into myofibroblasts sooner for a faster scar formation [51]. A TSP1 antagonist prevented TGFβ1-mediated cardiac fibrosis in diabetic rats [155]. In a pressure overload model of cardiomyopathy, TSP1 knockout mice displayed reduced TGFβ1-SMAD signaling with reduced myofibroblast differentiation and increased MMP9 and MMP3 activity, an efficient activator of MMP9 [156,157]. It was also found that TSP1 binds to pro-LOX, inhibiting its activation by BMP1 [52]. In vitro studies have shown that TSP1 can also directly bind to MMP2, and this interaction results in inhibition of the MMPs rather than proteolytic degradation of TSP1 by these MMPs [53]. On the other hand, interaction of TSP2 with MMP2 serves as a mechanism for clearance of MMP2 through endocytosis by lipoprotein receptor-related protein [54], similar to the mechanism proposed for clearance of the proMMP2-TIMP2 complex [55]. Consistent with the ability of TSP2 in clearing MMP2, and possibly other MMPs, TSP2-knock out mice exhibit increased activity of MMP2 and MMP9, which could be the underlying mechanism for the significantly higher rate of cardiac rupture following Ang II administration in these mice [56].

TSP3 and TSP4 share similarities in protein sequence and structure, and both have been found to be upregulated in cardiac disease [56,57,58,59]. Recently, TSP3 has been identified to augment injury-induced cardiomyopathy [60]. In a pressure overload model of heart disease, TSP3 overexpression resulted in greater hypertrophy, exacerbated ventricular remodeling and dilation, and greater cardiac fibrosis. Conversely, TSP3-deficiency protected the heart from cardiomyopathy and fibrosis following pressure overload. Lacking all TSPs except TSP3 (*Thbs1/2/4/5*^−/−^) resulted in mortality following pressure overload due to upregulation of TSP3 in these mice; whereas mice lacking all five TSPs showed 77% survival following pressure overload, which was attributed to the lack of TSP3 upregulation in these mice [60].

Despite sharing sequence and structural homology with TSP3, TSP4 was found to be cardioprotective following MI as well as pressure overload by inducing an adaptive ER stress response to protect the cardiomyocytes [62]. TSP4-deficient hearts exhibited increased fibrosis following cardiac pressure overload [61], with increased mRNA and protein levels of collagen I and III, to which TSP4 can directly bind [63]. Addition of recombinant TSP4 to fibroblasts in vitro reduced collagen production [61]. TSP4 acts in opposition to TSP1 and TSP2 by inhibiting profibrotic mechanisms. TSP5 has been understudied in regards to heart fibrosis, but like TSP1-4, is upregulated in cardiac disease [59].

SPARC (secreted protein, acidic and rich in cysteine) is a matricellular protein that is a potent modulator of cellular function, necessary for soluble procollagen processing into insoluble fibrillar collagen [46]. SPARC expression is increased in response to cardiac pressure overload, and has been found to be necessary for the development of myocardial fibrosis in this model [46]. SPARC expression is increased after MI and is spatially and temporally related to the fibrous scar formation [47,48]. SPARC-deficiency resulted in increased mortality and heart failure via disorganized granulation tissue formation and impaired scar maturation following myocardial infarction [158]. Fibroblasts and macrophages have been identified as main sources of SPARC in the myocardium [44,45]. Macrophage-derived SPARC was found to precede collagen deposition in myocardial fibrosis, which enhanced post-synthetic collagen processing, insoluble collagen content, and contributed to the development of fibrosis in a murine pressure overload model [49].

Periostin is another matricellular protein that is upregulated in fibrotic hearts, in activated myofibroblasts, and in the interstitial matrix. Absence of periostin decreased the recruitment of fibroblasts, impaired collagen fibrillogenesis, and increased the rate of cardiac rupture [64,65]. The specific mechanism of periostin function in cardiac fibrosis has not been elucidated yet, but it has been proposed to promote fibrosis in the lungs by inducing myofibroblasts differentiation [66]. Addition of periostin to cultured fibroblasts increased connective tissue growth factor (CTGF) and LOX mRNA expression [66]. Secretion of periostin in lung fibrosis is increased in response to interleukins, IL-4 and IL-13, and TGFβ1, all of which have been linked to fibrosis in cardiac remodeling [159,160,161].

Tenascin-C (TNC) is a matricellular protein that is induced in fibrotic hearts [69,70,71]. It is localized in areas with myofibroblast infiltration, and TNC loss demonstrated delayed myofibroblast recruitment to the injury site [67,68]. In an MI model, TNC-knockout mice had reduced diastolic dysfunction and cardiac remodeling associated with a reduction in myocardial fibrosis [72]. In contrast, TNC-deficiency in a cardiac pressure overload model resulted in exacerbated fibrosis, and deterioration of all functional LV parameters (ejection fraction, end-systolic, and end-diastolic dimensions, etc.) [73]. This study concluded that TNC in the bone marrow, and not the myocardium, protected the myocardium from excessive remodeling in response to cardiac pressure overload by controlling the inflammatory response.

## 6. Matricryptins Can Regulate Fibrosis

ECM is a renewable structure that undergoes continuous physiological remodeling, whereby the existing proteins are degraded by matrix metalloproteinases (MMPs) and are replaced by newly synthesized proteins. Proteolytic activity of the MMPs is kept in check by tissue inhibitors of metalloproteinases (TIMPs). In heart disease, an imbalance between MMPs and TIMPs is one of the many factors that contributes to the adverse ECM remodeling and fibrosis. The role of MMPs and TIMPs in heart disease has been reviewed elsewhere [6,77,162,163]. In addition to posing structural instability, ECM degradation results in generation of proteolytic fragments, called matricryptins that contain exposed matricryptic sites [164]. This term is only used in referring to biologically active fragments that contain a cryptic domain that is not normally exposed in the intact molecule (Table 3). Matricryptins such as endostatin and tumstatin are naturally occurring fragments of basement membrane (non-fibrillar) collagens XVIII and IV, respectively, and have been reported to possess biological functions such as anti-angiogenic effects, and are associated with various diseases including cardiovascular diseases [165,166]. However, the function of endostatin remains controversial. In a rat model of MI, endostatin inhibition worsened mortality, cardiac hypertrophy and fibrosis [167]. Anti-endostatin antibody increased fibrosis in the non-infarcted myocardium in a rat model [167], while endostatin-derived peptide (E4) prevented bleomycin-induced pulmonary fibrosis [168]. Meanwhile, pro-fibrotic effects of endostatin have also been reported through triggering of proliferation, migration, and wound healing in cardiac fibroblasts [169]. A novel collagen type I-α1 chain-derived matricryptin (p1158/59) has also been reported to regulate scar formation and myocardial remodeling following myocardial infarction [170].

Collagen IV, a non-fibrillar collagen and a predominant protein in myocardial basement membrane, has been identified to contain several matricryptins that are released through the proteolytic function of MMPs. These matricryptins include arresten (α1 chain, by MT1-MMP and MT2-MMP), canstatin (α2 chain, by MT1-MMP and MT2-MMP), tumstatin (α3 chain, by MMP9), tetrastatin (α4 chain), pentastatin (α5 chain) and hexastatin (α6 chain) [166]. A few matricryptins have been reported to impact fibroblast function and therefore are implicated in myocardial fibrosis. Canstatin has been reported to promote cardiac fibroblast migration [171], secretion of MMPs and inhibition of collagen gel contraction in myofibroblasts isolated from the infarcted myocardium from rats [172]. Tumstatin is significantly increased following cardiac pressure-overload, and has been reported to stimulate the proliferation and migration of cardiac fibroblasts, thereby suggested to play a pro-fibrotic role [173].

**Table 3:** List of Matricryptins with reported contribution to myocardial fibrosis.

Matricrptins	Expression in Fibrotic Heart Disease	Role in Fibrotic Heart Disease
p1158/59	Cleavage product of collagen 1α1 by MMP2, found in both human and mouse plasma post-MI [170].	A pro-angiogenic factor that promotes early ECM deposition post-MI, reducing adverse LV remodeling [170].
Arresten	Cleavage product of collagen IVα1 by MT1-MMP and MT2-MMP [166]. Expression is decreased 1-day post-MI in rats but increased in an I/R pig model [174,175].	An anti-angiogenic and pro-apoptotic molecule [176,177].
Canstatin	Cleavage product of collagen IVα2 by MT1-MMP and MT2-MMP [166]. Expression is decreased 1-day post-MI [174].	Stimulates the migration of rat cardiac fibroblasts through the increased expression of MMP2 [171].
Tumstatin	Cleavage product of collagen IVα3 by MMP9 [166]. Expression is increased after cardiac pressure overload [173].	Stimulates the proliferation and migration of cardiac fibroblasts [173].
Tetrastatin	Cleavage product of collagen IV α4 [166].	Unknown.
Pentastatin	Cleavage product of collagen IV α5 [166].	Unknown.
Hexastatin	Cleavage product of collagen IV α6 [166].	Unknown.
Endostatin	Cleavage product of collagen XVIII [166]. Expression increased in rats post-MI [167].	Anti-angiogenic but inhibition promotes adverse remodeling and fibrosis in rats post-MI [167]. Promotes proliferation, migration and wound healing in cardiac fibroblasts [169].
Endorepellin	Cleavage product of perlecan, a proteoglycan [178].	Anti-angiogenic and pro-fibrogenic [178].

Many matricryptins released from collagens (endostatin, arresten, constatin, tumstatin, restin, and vastatin) and from proteoglycans (endorepellin) correspond to the C-terminal domain of those molecules, and most exert their biological activities through signaling pathways mediated by integrins [179]. Various MMPs and cathepsins (B, L, S, and V) are involved in the release of matricryptins. MMP2 can degrade a number of ECM molecules, and appears to act as a main ‘decryptase’ with MT1-MMP for collagen IV and laminin [180]. MT1-MMP and MT2-MMP are involved in the release in of arresten [181] which is degraded by cathepsin S [182]. Interestingly, arresten and canstatin trigger transcriptional induction of MT2-MMP, collagen IV as well as pro-proliferative gene expression via integrin β1 signaling, therefore influencing collagen synthesis as well as proteolysis during morphogenesis.

Hyaluronidase and heparinase that degrade glycosaminoglycans also release matricryptins. A matricryptin can also be further processed as it has been reported for the C-terminal matricryptin of perlecan, endorepellin, which is cleaved by Bone Morphogenetic Protein-1 (BMP1) to generate the terminal laminin-like globular LG3 domain that possesses most of the biological activity on endothelial cells [183]. Endorepellin is a proteoglycan matricryptin, a fragment of perlecan with anti-apoptotic and pro-fibrogenic activity that is proteolyzed by cathepsin-L and is released to the extracellular space upon activation of caspase 3 [178]. Fragments of hyaluronan, a giant glycosaminoglycan (molecular weight of multi-million Dalton) can be generated by hyaluronidases 1 and 2 during tissue remodeling, or by reactive oxygen species at the site of tissue injury, collectively triggering tissue injury, inflammation, and ultimately fibrosis [184].

Most matricryptins transduce cell signaling by binding to cell surface receptors, most commonly to integrins, and some bind to heparan sulfate chains. Many of these integrins that regulate angiogenesis (α5β1, αvβ3, αvβ5) bind to heparan sulfate. In addition, binding of matricryptins to different cell types may be via different cell surface receptors. For instance, heparan sulfate proteoglycans could act as receptors or co-receptors for arresten [185]. However, most studies have focused on the signaling mediated by integrins. The anti-angiogenic [176] and pro-apoptotic [177] activities of arresten, both are mediated via integrin α1β1, but through different signaling pathways. Integrin α1β1 in fibroblasts [186] could mediate arresten effects on fibroblast proliferation or apoptosis. Therefore, proteolytic degradation of ECM proteins should not be superficially interpreted as reduced ECM content and less fibrosis, but it is critical to recognize that the ECM degradation products can further activate pro-fibrotic pathways and trigger fibrosis.

## 7. General Conclusions

It has become increasingly evident that the extracellular matrix is a complex and uniquely important component of the myocardial tissue whose function extends well beyond serving as a structural support network. ECM contributes to physical, physiological, biochemical, cellular, and pathological events in the heart. In addition to the fibrillar ECM, the numerous non-fibrillar proteins in the ECM each play significant and distinct functions in cardiac response to injury. Moreover, it is critical to note that ECM remodeling is a far more complex process than initially perceived. For instance, ECM degradation does not simply translate to reduced fibrosis, but degradation of ECM proteins can release latent growth factors and cytokines (sequestered in the ECM by proteoglycans), and/or generate matricryptins that further promote pro-fibrotic events. Non-fibrillar ECM proteins have emerged as important players in myocardial remodeling, however, much more needs to be unraveled on the specific and collective functions of the proteoglycans, glycoproteins and the basement membrane proteins in myocardial fibrosis, before successful development of anti-fibrosis therapies.

## Figures and Tables

**Table 1:** Fibrillar extracellular matrix (ECM) proteins in the heart that contribute to myocardial fibrosis.

Fibrillar Proteins	Expression in Fibrotic Heart Diseases	Role in Fibrotic Heart Disease
Collagen type I	Expression is increased in dilated myocardiopathy, ischemic heart disease and myocardial infarction, and in response to hypertension and pressure overload in both humans and animal models [5,6,7,8].	Deposited by activated fibroblasts [6]. Excessive collagen I deposition reduces heart compliance and result in increased dilation of the heart and can lead to heart failure [5,6]. In the ischemic heart, collagen I replaces necrotic cardiomyocytes to form the infart scar [6].
Collagen III	Expression is increased in all forms of fibrotic heart disease [6,7,8,9].	Deposited by activated fibroblasts [6]. Increased collagen III can reduce heart compliance and result in increased dilation of the heart [6,9].
Elastin	Degraded in rats and patients with acute myocardial ischemia [10,11]. During the compensatory phase of pressure overload, elastin expression is increased [12].	Degradation or mutation of elastin can lead to fibrotic heart disease (and aortic wall stiffening) [12,13]. Overexpression of elastin the infarct of ischemic hearts reduced LV dilation and infarct size, suggesting that preserving elastin is beneficial in fibrotic heart disease [14].

**Table 2:** Non-fibrillar ECM proteins that contribute to myocardial fibrosis.

	Expression in Fibrotic Heart Disease	Role in Fibrotic Heart Disease
Fibronectin (FN)	Expression is increased in hypertrophic rat hearts, and post-MI [15,16].	Enables the differentiation of cardiac fibroblasts to myofibroblasts to promote fibrosis [17]. Fibronectin promotes the maturation, stabilization and deposition of collagen I [18].
Syndecan-1	Increased expression in fibrotic areas of angiotensin II induced cardiac fibrosis [19]. Increased expression post-MI [20].	Promotes collagen upregulation, cross-linking and matrix formation [21].
Syndecan-4	Increased expression post-MI [20]. Found at focal adhesions, the site of mechanotransduction signaling [22].	Promotes myofibroblast differentiation and collagen expression [23,24]. Facilitates LOX collagen cross-linking activity [25].
Glypican-6 (GPC6)	Produced by cardiac fibroblasts and cardiomyocytes; upregulated with pressure overload and angiotensin II in mice [26].	In vitro experiments could not show that GPC6 has an effect on cardiac fibroblast collagen expression, proliferation, or migration [26].
Versican	Produced by cardiac fibroblasts and cardiomyocytes [27,28]. Upregulated in the hearts of pressure-overloaded rats [29].	Versican expression and cleavage was increased following stimulation of cardiac cells with cytokines associated with heart failure, indicating that versican is under inflammatory control [30].
Agrin and Perlecan	Found in the infarct and border regions post-MI [31].	Perlecan and agrin can bind to and accumulate growth factors (FGF, TGFβ, BMP, VEGF, HB-EGF) in the ECM through their heparin sulfate groups [32,33,34,35,36,37].
Biglycan	Expression is increased following MI or pressure overload [38].	A pro-fibrotic proteoglycan that co-localizes with and stabilizes collagen; necessary in wound healing following MI, but is detrimental following pressure overload through increased cardiac fibrosis [39,40].
Decorin	Expression is increased following MI or pressure overload [38].	Inhibits TGFβ1 activity by sequestering TGFβ1 (in latent form) and decreasing its expression [41]. Antifibrotic by inhibiting the TGFβ1-Smad pathway in hypertensive rats [42].
Lumican	Lumican is abundant in fibrotic tissues including the ischemic hearts [43]. Expression is increased in the hearts of HFrEF patients and pressure overloaded mice [38]. Expression is increased following mechanical stretch, IL-1β and LPS [38].	Addition of recombinant lumican in vitro increased collagen I and lysyl oxidase expression [38].
Secreted protein acidic and rich in cysteine (SPARC)	Secreted by fibroblasts and macrophages [44,45]. Expression is increased in response to pressure overload; is necessary for the development of myocardial fibrosis [46]. Expression is increased after MI and is spatially; temporally related to scar formation [47,48].	Necessary for processing soluble procollagen into insoluble fibrillar collagen [46]. Macrophage-derived SPARC contributes to development of fibrosis in a murine pressure overload model [49].
Thrombospondin-1 (TSP1)	Expression is strongly correlated to collagen expression in human explanted hearts [50].	Can activate TGFβ1 during the inflammatory stage of MI, allows fibroblasts to differentiate into myofibroblasts [51]. Binds to pro-LOX, inhibiting its activation by BMP1 [52]. TSP-1 can clear MMP2 and MMP9 via endocytosis to promote fibrosis [53,54,55].
Thrombospondin-2 (TSP2)	Expression is strongly correlated to collagen expression in human explanted hearts [50].	Promotes fibrotic deposition following angiotensin II infusion [56].
Thrombospondin-3 (TSP3)	Upregulated in cardiac disease [56,57,58,59].	Promotes greater hypertrophy, exacerbated ventricular remodeling and dilation, and greater cardiac fibrosis in a pressure overload model [60].
Thrombospondin-4 (TSP4)	Upregulated in cardiac disease [56,57,58,59].	TSP-4 acts in opposition to TSP-1 and -2 by inhibiting profibrotic mechanisms [61,62,63].
Periostin (PN)	Upregulated in fibrotic hearts and found in activated myofibroblasts and the interstitial matrix [64].	Contributes to recruitment of fibroblasts and collagen fibrillogenesis [64,65]. Addition of periostin in vitro to cultured fibroblasts increased connective tissue growth factor and LOX mRNA expression [66].
Tenascin-C (TNC)	Upregulated in fibrotic hearts and is localized in areas with activated myofibroblasts [67,68,69,70,71].	TNC negatively impacts post-MI remodeling, but is beneficial in attenuating fibrosis in a pressure overload model [72,73].
